# Performance of Copper as a Catalyst for Fenton-like Processes in Highly Saline Solutions

**DOI:** 10.3390/molecules30112298

**Published:** 2025-05-23

**Authors:** Xavier Orts, Jordi Arévalo, Antonio Arques, Ana M. Amat, Lucas Santos-Juanes

**Affiliations:** Advanced Oxidation Processes Group, Department of Textile and Paper Engineering, Universitat Politècnica de València, Plaza Ferrándiz y Carbonell 1, 03801 Alcoy, Spainaamat@txp.upv.es (A.M.A.)

**Keywords:** Fenton-like, copper, chlorides, seawater

## Abstract

The catalytic performance of copper in Fenton-like processes was investigated under conditions of elevated chloride concentrations. Model solutions were prepared containing four target pollutants (50 mg/L each), Cu (II) at 50 mg/L, and a stoichiometric dose of hydrogen peroxide sufficient for complete oxidation of the organic matter. Chloride levels ranged from low concentrations to those representative of both synthetic and natural seawater (36 g/L NaCl). An increase in chloride concentration consistently led to greater pollutant removal efficiency. The influence of pH on process performance was also assessed in saline and real seawater matrices. An optimal pH range between 6 and 7 was identified in both cases, where the reactivity of copper–chloride complexes was maximized while the formation of insoluble, catalytically inactive copper species was suppressed. Monitoring of pH, soluble copper concentration, and hydrogen peroxide consumption supported the conclusion that real seawater provides the most favorable conditions for copper–chloride catalyzed Fenton-like reactions. These results demonstrate the high potential of copper-based advanced oxidation processes in saline environments, particularly in applications where traditional methods exhibit limited efficiency.

## 1. Introduction

Advanced oxidation processes (AOPs) have gained significant attention in recent decades as powerful technologies for the degradation and mineralization of refractory organic pollutants. These processes rely on the in-situ generation of highly reactive oxygen species, mainly hydroxyl radicals (•OH), which exhibit non-selective oxidation capabilities and high redox potential, enabling the transformation of a wide spectrum of contaminants into harmless end-products [1]. Among the various AOPs developed, the Fenton and photo-Fenton processes have been extensively studied due to their efficiency and simplicity. The classical Fenton reaction is based on the catalytic decomposition of hydrogen peroxide (H_2_O_2_) by ferrous ions (Fe^2^⁺) in an acidic medium, generating hydroxyl radicals. In the photo-Fenton process, UV or visible light is used to accelerate the regeneration of Fe^2^⁺ from Fe^3^⁺, increasing the radical production rate and enhancing overall process efficiency [2,3].

However, the traditional Fenton process presents several limitations that restrict its practical application. Chief among these is the narrow pH range (typically around 2.8–3.0) required to maintain iron solubility and catalytic activity. At higher pH levels, iron tends to precipitate as ferric hydroxides, significantly reducing radical generation and thus treatment efficiency. Moreover, iron-based Fenton reactions can be adversely affected by the presence of common anions such as chloride or carbonate, which alter iron speciation and act as radical scavengers, reducing the availability of •OH and promoting the formation of less reactive species [4,5,6].

To overcome these drawbacks, increasing attention has been paid to the use of alternative transition metals in Fenton-like systems. Chromium, copper, aluminium or cobalt combined with hydrogen peroxide or other oxidants are able to perform a catalytic redox cycle equivalent to the traditional Fenton [7].

Among them, copper has emerged as a particularly promising catalyst, offering several advantages over iron. Copper can operate over a wider pH range, remaining soluble and catalytically active up to pH 6, which allows for more flexible treatment conditions and avoids the need for strong acidification [8,9]. Furthermore, copper-based Fenton-like systems follow a redox cycle similar to the classical process (Cu^2^⁺/Cu⁺), generating hydroxyl and hydroperoxyl radicals through reactions with H_2_O_2_:

Cu^2+^ + H_2_O_2_ → Cu^+^ + HO_2_^•^ + H^+^ (1)

Cu^+^ + H_2_O_2_ → Cu^2+^ + HO^•^ + HO^−^ (2)

However, Cu⁺ can also undergo oxidation by dissolved oxygen, regenerating Cu^2^⁺ without producing radicals, which necessitates higher hydrogen peroxide dosages to compensate for the following side reaction [7]:

4Cu^+^ + 4H^+^ + O_2_ → 4Cu^2+^ + 2H_2_O (3)

Despite this limitation, one of the most compelling reasons to consider copper as a Fenton-like catalyst is its positive interaction with chloride ions, particularly relevant in the context of saline wastewaters. Unlike iron, whose catalytic performance is often inhibited by chloride due to complexation and radical quenching, copper forms stable and catalytically active copper–chloride complexes (e.g., CuCl⁺, CuCl_2_^−^) that enhance degradation efficiency even at near-neutral pH values [10]. This distinct behavior gives copper-based systems a strategic advantage in treating high-salinity effluents such as seawater, desalination brines, or wastewaters from the agro-food, petroleum, textile, and leather industries [11,12,13,14].

In general, the presence of chlorides in advanced oxidation processes has been described as negative. For example, the UV/H_2_O_2_ process suffers a decrease in the degradation rate of phenol of approximately 70% in seawater [15], while the ozonation of nitrobenzene is inhibited by approximately 80% in the presence of high concentrations of chlorides [16]. The presence of chlorides in traditional acidic Fenton or photo-Fenton has been described as undesirable since the reaction rates of the pollutants removal suffer important decreases (between 40 and 50%) compared with those obtained in the absence of chlorides [4].

While copper is generally considered more toxic and expensive than iron, its presence as a pollutant in industrial wastewaters, particularly from metal plating, electronics manufacturing, or etching processes, presents an opportunity for resource recovery and circular economy implementation. Copper concentrations in these effluents can reach from tens of mg/L to several g/L [17,18,19,20], making them suitable not only for remediation but also as intrinsic sources of catalyst in Fenton-like processes. This dual role—catalyst and contaminant—supports the “waste-treats-waste” philosophy, promoting sustainable and cost-effective treatment strategies.

Although the behavior of copper as a Fenton-like catalyst in highly saline media seems very promising, copper presents some disadvantages compared to iron: it is expensive and toxic. For these reasons, the external addition of copper salts to perform a Fenton-type process does not seem to be a convenient solution unless it is used in low concentrations. However, dissolved copper salts can be found on some industrial effluents like metal finishing or etching industries in concentrations that range from tens of milligrams per liter to various grams per liter [18,19,20,21]. Therefore, the use of copper containing effluents as a source of copper for the treatment of highly saline wastewaters can be an option that is part of the principles of the circular economy and the motto “waste cleaning waste”.

With this background in mind, the present study aims to evaluate the applicability and efficiency of copper-catalyzed Fenton-like processes in the presence of chlorides, using both simulated and real seawater. To simulate an industrially relevant scenario, a mixture of four model contaminants was selected: amoxicillin, carbamazepine, caffeine, and acetaminophen—representative pharmaceuticals and personal care products frequently detected in aquatic environments. Their importance as contaminants in the environment has even led to the study of new specific sensors for their determination [22,23]. These compounds are known to be recalcitrant to conventional treatments and exhibit limited capacity to complex transition metals [24], which allows for a clearer assessment of the catalytic activity of copper.

The organic charge was high to represent a possible industrial wastewater with four model pollutants at 50 mg/L each. Given the prospect of using copper-laden effluents as a catalyst source, the system was tested at relatively high catalyst concentrations (50 mg/L Cu), and the effect of varying chloride levels was systematically studied. The effect of chlorides was studied in a wide range of concentrations including simulated and real seawater.

The degradation of the pollutants, mineralization of organic carbon, consumption of hydrogen peroxide, and evolution of copper species in solution were monitored to elucidate the mechanisms of the process. The findings contribute to the growing body of evidence supporting copper-based Fenton-like systems as viable, robust, and sustainable alternatives for treating saline and complex wastewater matrices, especially when aligned with circular economy principles.

## 2. Results and Discussion

### 2.1. Effect of NaCl Concentration and pH Changes

A first set of experiments was carried out modifying the concentration of chlorides in solution expressed as NaCl concentration. These values ranged from 0 to 36 g/L employing distilled water as a solvent, and finally, the experiments were performed with real seawater from the Mediterranean Sea with a NaCl concentration of about 36 g/L. The copper (II) concentration was fixed at 50 mg/L and the operation pH was adjusted to 6 at the beginning, without further modification, in all samples. Hydrogen peroxide was added at a concentration of 1000 mg/L which corresponds with the stochiometric concentration calculated to produce the complete oxidation of the pollutant’s mixture (50 mg/L of acetaminophen, amoxicillin, carbamazepine, and caffeine with an initial organic carbon concentration of 132 mg/L). Samples were periodically collected, and the results are presented as the evolution of the sum of concentrations (only for selected conditions) and as the percentage of total degradation after one hour of reaction for all tested conditions. Additionally, the mineralization of the organic matter present was monitored and reported as a percentage in all experiments. The evolution of the pollutant’s concentration represented individually can be consulted in the Supplementary Information. In all cases, caffeine was the most reluctant pollutant to be degraded, while acetaminophen, amoxicillin, and carbamazepine showed similar behavior.

**Figure 1:**
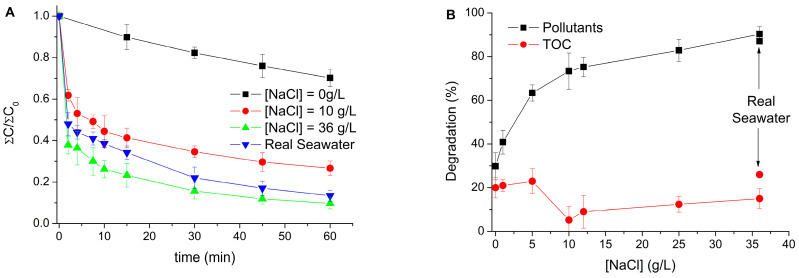
Different saline conditions effects on the sum of the pollutant’s degradation (**A**), and in the final percentage of pollutants degradation and mineralization (**B**). Experimental conditions: 50 mg/L of acetaminophen, amoxicillin, carbamazepine, and caffeine; 50 mg/L of copper; 1000 mg/L (stochiometric amount) of hydrogen peroxide; initial pH of 6.

For the sake of clarity, only four conditions are presented in Figure 1A: the absence of chlorides, chlorides at concentrations of 10 and 36 g/L, and real seawater as matrix. The total concentration was calculated as follows: the concentration of each compound in each sample was determined and then summed. The resulting values were normalized using the initial total concentration. The degradation of the mixture of pollutants was fast in the first stages of the process and the increase in chlorides concentration produced an increase in the initial rate of degradation and in the final degradation achieved. All final degradations achieved in every experimental condition are presented in Figure 1B. The degradation achieved was always higher when the presence of chlorides was increased. Thus, a continuous improvement of the degradation achieved was observed for the concentrations 0, 1, 2, 5, 10, 12, 25, and 36 g/L, and the experiments carried out with real seawater obtained results quite similar to those with 36 g/L. Contrary to the effect produced in other advanced oxidation processes, it seemed that an increase in chlorides concentration increased the efficiency of the pollutants degradation. However, the mineralization behavior did not follow a clear tendency.

As a Fenton-like process, the speciation of the metal in solution and its solubility in water are the factors that mainly rule the process. Copper salts begin to form inactive insoluble species in a water solution (mainly Cu(OH)_2_) at pH values around 6.0 with a solubility constant at pH 7 K_sp_ = 10^−19.32^ that allows concentration values lower than 1 mg/L [9]. However, chlorides can form different soluble copper complexes like CuCl^2−^ or CuCl_3_^2−^ with increasing chloride content by increasing the number of chloride ligands available [25,26]. Finally, the activity of copper–chloride complexes to enhance redox reactivity in Fenton-like processes was previously reported and the reactivity of the Cu (II) complex towards H_2_O_2_ increased markedly with increasing coordination number [10]. Therefore, it seemed reasonable that at higher chloride concentrations the more coordinated complexes were promoted with the high reactivity in Fenton-like processes obtaining higher degradation of the pollutant’s mixture at higher NaCl concentrations.

To explain the different trends between pollutants degradation and TOC mineralization it is necessary to consider the different species responsible of the oxidative process and its prevalence under different conditions. In the absence of chlorides (0 g/L conditions), copper salts can react with hydrogen peroxide (reactions 1 and 2) to produce reactive oxygen species (HO_2_^•^ and HO^•^) with high reactivity with organic matter and therefore, are able to produce pollutant oxidation but also organic matter mineralization [7]. However, at the working pH of 6, part of the dissolved copper starts to form inactive insoluble species and thus, gradually the available copper in the solution suffers an important decrease. Because of the scarce stability of the catalyst and the high reactivity of radicals formed, a low degradation of pollutants was achieved but with a high mineralization percentage.

The behavior of the system started to change by increasing the presence of chlorides in the media that allowed forming copper complexes. Copper complexes also react with hydrogen peroxide to form reactive oxygen species following the equations described below [27]:

Cu (II)-Cl_n_ + H_2_O_2_ → Cu (I)-Cl_n_ + O_2_^•−^ + 2H^+^ (4)

Cu (I)-Cl_n_ + H_2_O_2_ → Cu (II)-Cl_n_ + HO^•^ + HO^−^ (5)

Chloride–copper complexes present higher solubility at neutral pH values and higher reactivity for the reductive step of copper (II) to form copper (I) than those obtained for copper alone. Consequently, the amount of copper acting as catalyst remains higher in the presence of chlorides and increasing its amount ensures the formation of these complexes. However, the scavenging effect of chlorides among the reactive radicals to form the corresponding chloride radicals (Cl^•^ and Cl_2_^•−^) also increases being the main responsibility of organic matter oxidation [28,29].

HO^•^ + Cl^−^ → HOCl^•−^ (6)

HOCl^•−^ + H^+^ → Cl^•^ + H_2_O (7)

Cl^• ^ +  Cl^−^  → Cl_2_^•−^ (8)

Considering that chloride radicals have an oxidative potential lower than reactive oxygen radicals but their lifetime is about 1000 times higher [25], the results obtained were expected: high degradation of pollutants but less mineralization of the organic matter.

It is interesting to compare the results obtained with 36 g/L of sodium chloride with those obtained with real seawater. Although the amount of sodium chloride in the Mediterranean Sea is about 36 g/L, there are other species than can interact with copper such as fluorides, borates, bicarbonates, or sulfates [30]. Among all of them, the buffer effect produced by bicarbonates could maintain the pH stable during the process. Thus, while experiments with distilled water had a pH value at the end of the reaction close to 4.5, in seawater this value remained around 6.5. This fact that was very important as it will be explained in the next sections.

**Figure 2:**
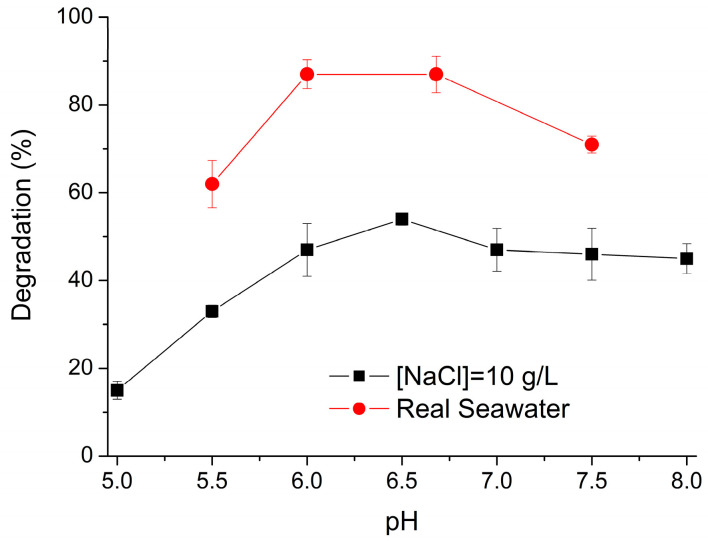
Degradation percentages achieved of the pollutant’s mixture at different pH values in different water matrices. Experimental conditions: 50 mg/L of acetaminophen, amoxicillin, carbamazepine and caffeine; 50 mg/L of copper; 1000 mg/L (stochiometric amount) of hydrogen peroxide.

The effect of the chloride’s concentration was tested at the same initial pH and was kept free during the experiments. In order to find the optimum pH value for this process, the experiments were repeated at the fixed value of a NaCl concentration of 10 g/L in distilled water changing the initial pH from 5 to 8, and the same effect was studied with real seawater. As shown in Figure 2, there was a maximum degradation of the mixture of pollutants between 6 and 7 in both water matrices and it has to be highlighted that the natural pH of seawater (after the addition of pollutants and copper) was around 6.7, which corresponds to a value inside the optimum zone of work. At this point, it should be clarified that the original pH of seawater was 8.1 but the addition of the pollutants and the copper salts produced a decrease in this value.

As seen in Figure 2, the efficiency of the process clearly decreased at pH values lower than 6 while at higher than 7, the efficiency also showed a slight decrease, proving that the Fenton-like process employing copper as catalyst in saline matrices can be applied at neutral conditions.

The reasons to explain this behavior with pH were again related to the speciation and solubility of copper, but also with the copper (II) reduction at different pH values. As commented before, the precipitation of copper starts at pH 6.0 and becomes more relevant at higher pH values, so lower values should be more convenient. However, copper chloride complexes are less predominant at lower pH values where copper activity in the Fenton process is drastically reduced [27]. On the other hand, the reductive step to convert Cu (II) into Cu (I) (kinetically limiting) is more favorable at pH 8 than at pH 6 but the formation of insoluble copper species is also favored [28]. For these reasons, the optimum range to operate the process is from 6 to 7 where the opposite effects seem to be balanced.

**Scheme 1:**
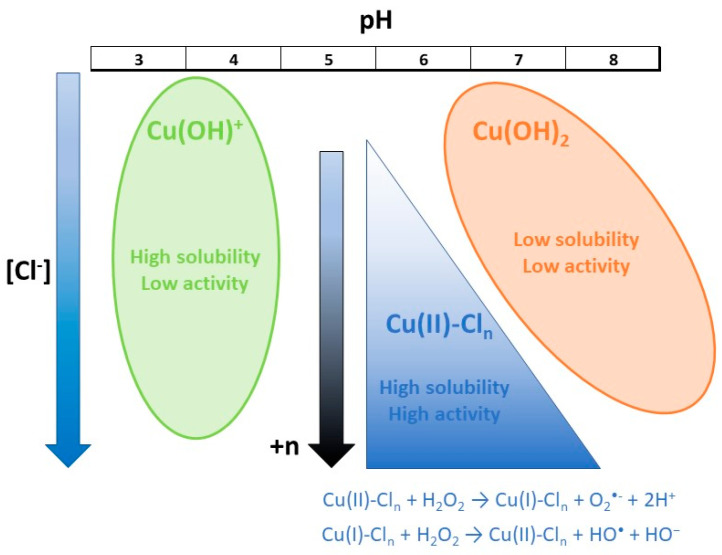
Different speciation of copper in solution at different pH values, chlorine concentrations, and active zones for Fenton-like processes.

Scheme 1 summarizes all the effects involved in the Fenton-type process. First, it should be noted that at pH values close to 6, copper begins to form copper hydroxide, which is insoluble and has no photocatalytic activity. Chlorides can compete with the formation of copper hydroxide by forming chlorine complexes that are soluble and active in the presence of hydrogen peroxide (reactions 4 and 5). Increasing chloride concentration favors the formation of complexes with greater coordination (+n), which are more stable and more efficient in copper reduction. At pH values below 5, the formation of chlorinated copper complexes is limited, and copper aqueous complexes predominate. These complexes are soluble but have little activity in the presence of hydrogen peroxide since they react readily with the oxygen present, inhibiting the formation of radicals (reactions 1 to 3).

### 2.2. Evolution of Copper in Solution

The main reason pointed to in the previous section to explain the behavior of Figure 1 was the changes in the copper speciation in the presence of chlorides. The formation of active chloride complexes avoided copper precipitation and allowed the redox cycle of copper more efficiently than in the absence of chlorides. To evaluate the precipitation of copper at the working conditions (initial pH of 6 without further control), its concentration was measured during the process of four selected conditions and in all cases at the end of the process.

**Figure 3:**
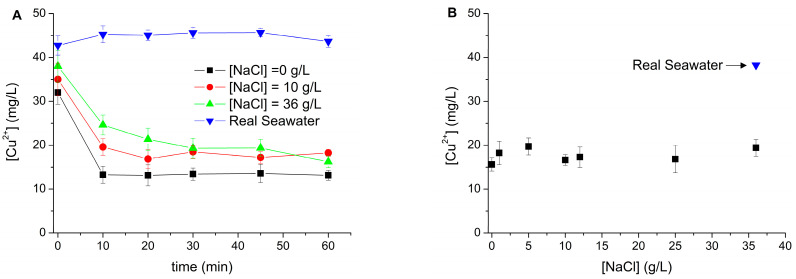
Evolution of copper concentration during the process of four selected conditions (**A**). Amount of copper in solution after 1-h reaction at different values of sodium chloride concentration and with real seawater (**B**). Experimental conditions: 50 mg/L of acetaminophen, amoxicillin, carbamazepine, and caffeine; 50 mg/L of copper; 1000 mg/L (stochiometric amount) of hydrogen peroxide.

All processes carried out with de-ionized water matrices obtained similar results as can be seen in Figure 3B. After a 1 h reaction, the remaining copper in the solution was close to 20 mg/L, thus more than 50% of the catalyst was lost by precipitation. This precipitation stopped at a certain value where the pH became low enough to prevent further precipitation. In all cases, the final value of pH was around 4.5, where the precipitation of copper stopped but the activity of copper as a Fenton-like catalyst was very low to continue with pollutant degradation, as shown in Figure 2. Although the behavior of copper in solution was very similar in all cases, the degradation of pollutants achieved (showed in Figure 1B) at increasing concentrations of sodium chloride were very different. This behavior can be explained by the fact that the time required to cause the copper precipitation and the consequent drop in pH value increases with the presence of chlorides that form the corresponding complexes. Furthermore, an increase in the chloride concentration shifts the equilibrium towards the formation of the more active complexes that react faster, as was discussed in Section 2.1.

Results obtained with real seawater showed that the amount of copper in the solution remained very high, close to 40 mg/L, at the end of the reaction with a final pH value that also remained almost constant (as discussed in next section). Real seawater presents a variety of inorganic anions that could form copper complexes apart from chlorides; sulfates, bicarbonates, bromides, borates, or fluorides decreasing its precipitation rate as copper hydroxide. For example, the formation of active complexes of copper with bicarbonates was previously described [31] and the presence of natural organic matter such as humic acid could also form copper complexes slowing its precipitation [32].

To account for potential problems with discharging dissolved copper into the environment, the amount of copper remaining after one day was assessed. In cases where distilled water was used, this value remained almost constant (around 20 mg/L) due to the pH drop to around 4.5 where copper is highly soluble. When real seawater was used, the values obtained after one day were less than 5 mg/L, since the pH remains stable (6–7) and copper tends to precipitate slowly.

Therefore, pH adjustment (if necessary) and subsequent decantation may be sufficient to remove the precipitated copper and keep discharges below the legal limits.

### 2.3. Processes with Simulated and Real Seawater

The experiments of the previous section showed an increase in the efficiency of pollutants degradation as the presence of chlorides in the medium increased. The highest value of chlorides was represented by the simulated and real seawater with a NaCl concentration close to 36 g/L. It was also assessed that the natural pH of real seawater (after the addition of the pollutants and catalyst) was optimal to carry out the process; therefore, it seems that the applications of copper-mediated Fenton-like processes will be of special interest in real seawater.

To gain further insight about the behavior of the process with this water matrix, new experiments were performed monitoring the evolution of pH during the oxidative process. As commented before, the optimum pH to apply the process ranges between 6 and 7, but in these experiments, the pH was adjusted at the beginning and kept free during the process. In the new experiments, the evolution of pH was monitored to measure possible variations during the process and to study the possibility of keeping this value constant by the addition of acidic or basic solutions.

**Figure 4:**
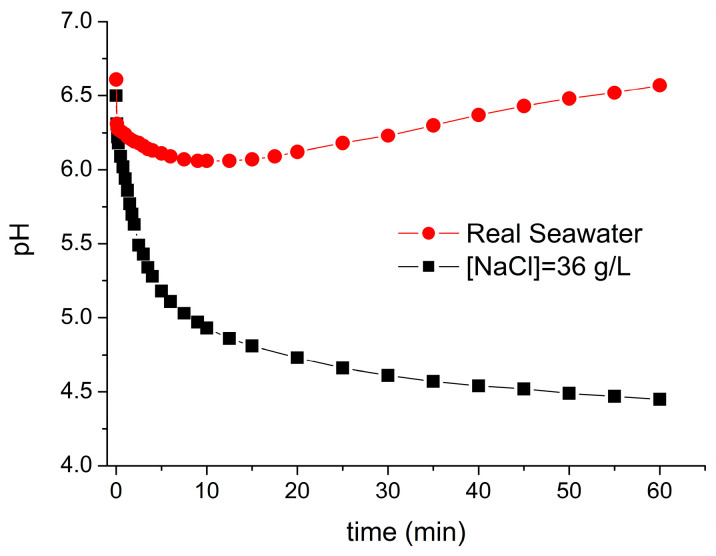
Evolution of pH during the oxidative process with real and simulated seawater. Experimental conditions: 50 mg/L of acetaminophen, amoxicillin, carbamazepine, and caffeine; 50 mg/L of copper; 1000 mg/L (stochiometric amount) of hydrogen peroxide.

As shown in Figure 4, there was a small variation on pH values with real seawater and an important drop of pH values was observed when simulated seawater was used. The main process that produced the fall in the pH was the formation of insoluble copper hydroxide as it has been explained in the previous section. The possible formation of carboxylic acids as transformation byproducts could also produce a decrease in pH values, but its contribution should be less relevant compared to copper precipitation. This variation of the pH becomes important in the absence of buffer solutions as occuring in simulated seawater. However, the presence of the buffer carbonate/bicarbonate in real seawater, with a measured inorganic carbon dose of 30 mg/L, prevented the drop in the pH values, and therefore, the variation on pH during the process was low.

Once the variation of pH during the oxidative process was monitored, the same experiment was carried out controlling the pH (value of 6.5) in the simulated seawater (36 g/L NaCl) to avoid variations higher than 0.1. The final degradation achieved was notably enhanced since the process was running during the entire reaction time at its optimum pH value.

**Figure 5:**
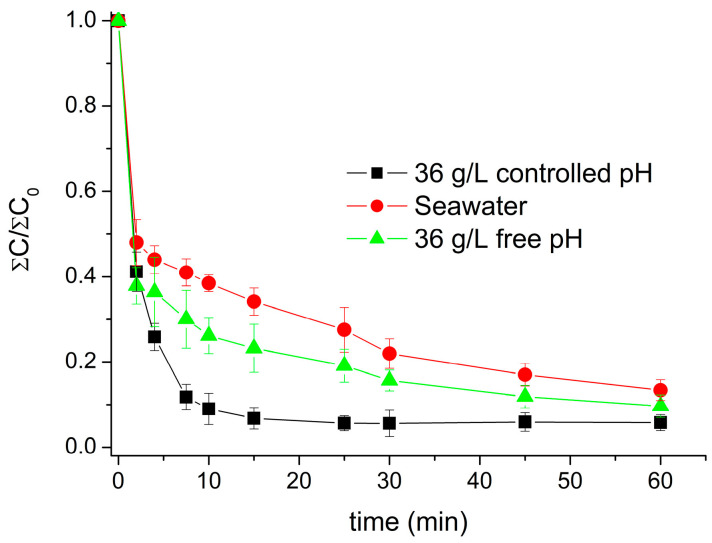
Evolution of the pollutant’s degradation for real seawater and for 36 g/L of NaCl water (simulated seawater) with free and controlled pH. Experimental conditions: 50 mg/L of acetaminophen, amoxicillin, carbamazepine and caffeine; 50 mg/L of copper; 1000 mg/L (stochiometric amount) of hydrogen peroxide.

Figure 5 shows the comparison of real seawater and simulated seawater when the pH was left free and when the pH was controlled. The positive effect of operating at an optimal pH throughout the entire process can be clearly seen in the degradation values achieved after 15 min of reaction. For this reaction time, the degradation achieved at a free pH is close to 75%, while at a controlled pH, this value rises above 90%.

The positive effect of a buffered media in real seawater was also observed in Figure 1, where the mineralization achieved increased compared with that obtained with 36 g/L sodium chloride in de-ionized water. While the degradation of pollutants was similar due to its fast degradation, the mineralization process that requires longer times was affected by the changes in pH.

**Figure 6:**
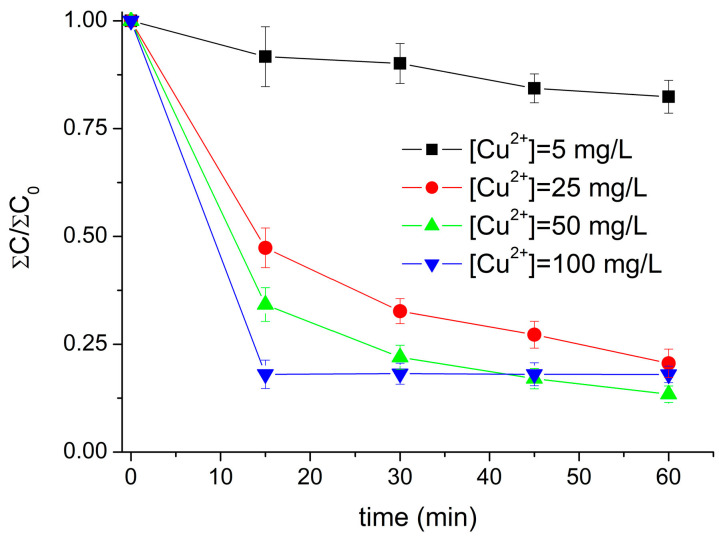
Concentration evolution of the pollutant’s degradation employing different amounts of copper in solution in a real seawater matrix. Experimental conditions: 50 mg/L of acetaminophen, amoxicillin, carbamazepine, and caffeine; 10 g/L of NaCl; 1000 mg/L (stochiometric amount) of hydrogen peroxide.

Since the degradation of the pollutants in real seawater was very fast and the presence of a buffered media preserves its activity, other amounts of copper in solution were tested to study the convenience of using lower amounts of the catalyst. Two values of concentration below the reference employed in all previous experiments (50 mg/L) and one value with concentration above the reference were performed in the same conditions of pollutants and hydrogen peroxide concentrations in real seawater at its natural pH. The evolution of the pollutants concentration during the experiment were represented as the sum of all of them, as can be seen in Figure 6.

The lowest copper concentration studied was 5 mg/L and we were only able to remove 20% of the pollutants after one hour of reaction. When 25 mg/L was employed, results obtained were significantly enhanced achieving 50% degradation in only 15 min with a final degradation of 75%. The reference value of 50 mg/L, employed in all previous experiments, was slightly faster in the degradation of pollutants and also achieved a higher final degradation value, close to 90%. The typical asymptotic behavior on initial degradation rates and total degradation was confirmed when 100 mg/L was employed and achieved low improvements with respect to those obtained with 50 mg/L. It should be mentioned that when 100 mg/L of copper was employed, all hydrogen peroxide present was consumed and the reaction stopped. Likely, the reactions between radicals formed and the hydrogen peroxide was responsible for this fast-inefficient consumption, as has been described in Fenton processes with high amounts of catalysts and oxidants [33].

With these results, the use of 25 mg/L of the catalyst seems to be the most efficient option since the conversion at short times is very high and the final value achieved after 1 h of reaction is very close to those obtained with a higher catalyst concentration.

### 2.4. Evolution of Hydrogen Peroxide Concentration

Hydrogen peroxide decomposes in Fenton-like processes following two mechanisms: reaction with the catalyst (desired consumption) and reaction with radicals (undesired consumption) [34]. In both cases, the evolution of hydrogen peroxide concentration in a Fenton-like process is indicative of the activity of the catalyst, and the inactivation of the catalyst undergoes a decrease of hydrogen peroxide consumption.

In previous sections, the importance of copper precipitation and pH changes in the solution have been described. The inactivation of the catalyst by formation of insoluble species and the modification of pH values that cause a loss in the activity of copper caused a decrease in the efficiency on the oxidative process. To link these effects with hydrogen peroxide consumption, the processes with simulated seawater and real seawater were repeated at free and controlled pH, and the consumption of hydrogen peroxide was monitored by the ammonium metavanadate colorimetric method described in Section 3.3. It is important to remember that the hydrogen peroxide was added to achieve the stochiometric concentration able to produce the complete oxidation of all the organic matter present, about 800 mg/L.

**Figure 7:**
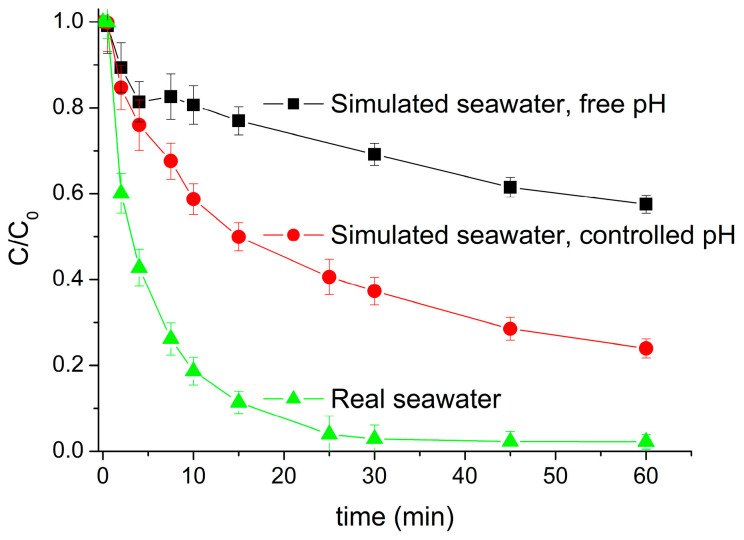
Hydrogen peroxide consumption under different water matrixes. Experimental conditions: 50 mg/L of acetaminophen, amoxicillin, carbamazepine, and caffeine; 50 mg/L of copper; 1000 mg/L (stochiometric amount) of hydrogen peroxide.

To establish a reference, the experiment carried out in the absence of sodium chloride was also repeated and the hydrogen peroxide evolution showed that at 15 min of reaction, the consumption stopped and remained constant at a value of 7.5% of the total amount (See Figure 7).

The evolution of hydroxide peroxide with simulated seawater with a NaCl concentration of 36 g/L showed the importance of controlling the pH. As showed in Figure 4, the sample with free pH suffered a decay in this value leaving the optimum working zone of pH determined in Figure 2. As a consequence, the activity of copper in solution was reduced and the consumption of hydrogen peroxide slowed after 5 min of fast consumption. In the experiment where pH was controlled, the hydrogen peroxide consumption remained high during the entire process, achieving a final consumption of nearly 80%, almost twice the value obtained in the experiment with free pH. These differences in hydrogen peroxide consumption were reflected in the differences on degradation shown in Figure 5.

Since the real seawater did not suffer important changes in its pH, probably due to the buffer effect of carbonates, there were no changes in the hydrogen peroxide rate of consumption being fast compared to those obtained with simulated seawater. In addition to the effect of maintaining the pH in an optimum zone of work, the amount of copper available was higher as showed in Figure 3B.

## 3. Materials and Methods

### 3.1. Materials and Reactor

Target contaminants were dissolved in a distilled water matrix produced from a Millipore water purifier. The model pollutants employed were high purity (>99%) acetaminophen, amoxicillin, carbamazepine, and caffeine, obtained from SIGMA-ALDRICH (Radnor, PA, USA).

The oxidant chosen for the Fenton-like reaction was the hydrogen peroxide (33% *w*/*v*) purchased by PanReac (Barcelona, Spain). The copper ion source was copper (II) sulphate 5-hydrate supplied also by PanReac. Saline conditions were achieved by adding sodium chloride manufactured by PanReac and using real seawater from the coast of Alicante, Spain, where water salinity is near 36 g/L.

The control of pH in each essay were done using 0.1 M solutions prepared from sodium hydroxide (99%) and sulfuric acid (96%), both supplied by PanReac. In order to study the process at each point, the reactions were stopped before their analysis. For this purpose, methanol supplied by PanReac was used with a purity of 99.9%.

The reactor was operated as a discontinued stirred tank reactor made of 250 mL cylindrical Pyrex vessel with a diameter of 6.5 cm and a height of 9.5 cm. For the mixing, an Ultraflat Magnetic Stirrer from Avantor (Saint Louis, MI, USA) was used at 500 rpm.

### 3.2. Experimental Method

All the contaminants were added at concentrations of 50 mg/L each. The solution was prepared one day before, and temperature and ultrasound were used in order to ensure a homogeneous solution for proper results. Sodium chloride was also added in this step to achieve the concentration needed in each experiment.

When the solution was ready, the copper (II) sulphate was added to achieve a 50 mg/L concentration of copper ions; then, the pH was adjusted to the objective value. Finally, the hydrogen peroxide was added to the solution at stoichiometric concentration to achieve the complete oxidation of the pollutants; 1000 mg/L.

The reactions were carried out in open cylindrical reactors with 250 mL of solution, under magnetic agitation (~400 rpm), at room temperature (~20 °C) without irradiation.

To follow the evolution of hydrogen peroxide, pollutant or copper concentration samples were taken periodically. To prevent the reaction during the storage before the analysis, 1 mL of the sample was mixed with 0.5 mL of methanol. It is well-known that the presence of methanol inhibits hydroxyl radical-initiated reactions.

All experiments were performed in duplicate at minimum.

### 3.3. Equipment and Analysis

The evolution of the pollutants in the course of the experiment was studied by the high-performance liquid chromatography method. The equipment used was reverse-phase column chromatograph, the model was the High-Performance Liquid Chromatograph Chromaster supplied by Hitachi (Tokyo, Japan). The eluents employed consisted in acid formic 10 mM supplied by PROLABO (Tokyo, Japan) and acetonitrile grade UHPL supplied by PanReac. The gradient used started with 97% of formic acid and 3% of acetonitrile until 100% of acetonitrile after 26 min, using a flow of 1 mL/min. The temperature was also fixed using the oven at 35 °C and the samples were analyzed at wavelength of 225 nm where all the pollutants could be analyzed clearly.

Dissolved organic carbon (DOC) and inorganic carbon (IC) was determined with a Shimadzu model TOC-V CSH apparatus (Kyoto, Japan). Before analysis, samples were filtered through polypropylene (VWR, Leicestershire, UK, 0.45 μm).

The evolution of hydrogen peroxide throughout the reaction was followed using peroxide indicator strips using the scale suitable for each mode. These strips were supplied by Labbox (Brussels, Belgium). For a fine determination of hydrogen peroxide consumption, a sample volume between 1 and 8 mL (depending on the expected concentration) was used, 1030 microliters of ammonium metavanadate solution (0.06 M ammonium metavanadate in 0.36 M sulfuric acid) were added and the flask, and filled to a volume of 10 mL with distilled water. The absorbance was measured at 450 nm.

The analysis of copper in the solution was determined using the thiosemicarbazone of 2-carboxybenzaldehyde (2CBTSC) method [35]. This method measures the yellow color formed by the union of copper and 2CBTSC with photometric estimation at 346 nm and has a detection limit of 0.5 mg/L.

To make the absorbance measurements, a Hitachi UH 5300 spectrophotometer (Tokyo, Japan) equipped with double beam was employed. Quartz cuvettes purchased by Akralab (Alicante, Spain) were used in all analyses.

## 4. Conclusions

Copper has proven to be a highly effective catalyst in Fenton-like processes under saline conditions, primarily due to the formation of active copper–chloride complexes (CuCl_n_), whose catalytic activity increases with chloride concentration. This interaction significantly enhances pollutant removal efficiency, although mineralization levels are somewhat limited, likely due to the formation of chlorine-based radicals, which are effective at oxidizing pollutants but less efficient in achieving complete organic matter degradation.

An optimal operating pH between 6 and 7 was identified, where copper complexes maintain high catalytic activity without substantial formation of inactive, insoluble species. Maintaining this pH range was shown to be crucial for preserving process performance, as evidenced by hydrogen peroxide consumption patterns under both controlled and uncontrolled pH conditions.

The use of real seawater as a reaction matrix further improved the process, as its natural buffering capacity helped sustain the pH within the optimal zone, allowing copper complexes to remain active. Additional beneficial effects observed in real seawater—potentially related to the presence of carbonates and dissolved organic matter acting as copper ligands—warrant further investigation.

Overall, the excellent performance of copper in high-chloride environments highlight its strong potential for advanced treatment of highly saline industrial wastewaters, reverse osmosis brines, and contaminated seawater, significantly expanding the applicability of Fenton-like processes in complex saline matrices.

## Data Availability

Data are contained within the article and Supplementary Materials.

## Supplementary Materials

The following supporting information can be downloaded at: https://www.mdpi.com/article/10.3390/molecules30112298/s1, Figure S1: Evolution of the of the pollutants degradation with reaction time. Experimental conditions: 0 g/L sodium chloride, 50 mg/L of acetaminophen, amoxicillin, carbamazepine and caffeine; 50 mg/L of copper; 1000 mg/L (stochiometric amount) of hydrogen peroxide; initial pH of 6; Figure S2: Evolution of the of the pollutants degradation with reaction time. Experimental conditions: 1 g/L sodium chloride, 50 mg/L of acetaminophen, amoxicillin, carbamazepine and caffeine; 50 mg/L of copper; 1000 mg/L (stochiometric amount) of hydrogen peroxide; initial pH of 6; Figure S3: Evolution of the of the pollutants degradation with reaction time. Experimental conditions: 10 g/L sodium chloride, 50 mg/L of acetaminophen, amoxicillin, carbamazepine and caffeine; 50 mg/L of copper; 1000 mg/L (stochiometric amount) of hydrogen peroxide; initial pH of 6; Figure S4: Evolution of the of the pollutants degradation with reaction time. Experimental conditions: 36 g/L sodium chloride, 50 mg/L of acetaminophen, amoxicillin, carbamazepine and caffeine; 50 mg/L of copper; 1000 mg/L (stochiometric amount) of hydrogen peroxide; initial pH of 6.
