# Supplementary material for: Performance of Copper as a Catalyst for Fenton-like Processes in Highly Saline Solutions
Source: Molecules. 2025 May 23;30(11):2298. doi: 10.3390/molecules30112298 (PMC12156137; doi:10.3390/molecules30112298)
Supplement: Supplementary file 1 [file molecules-30-02298-s001.zip › molecules-3596879-supplementary.pdf]

Supplementary Information

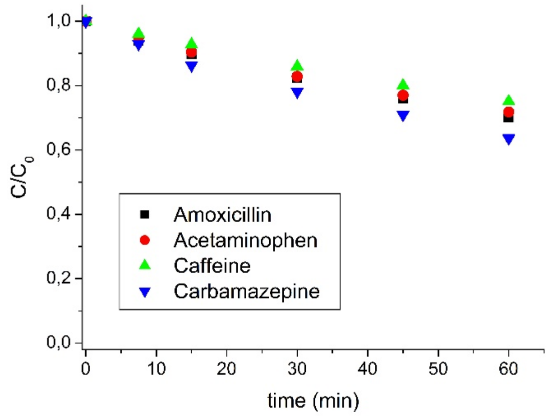

Figure S1. Evolution of the of the pollutants degradation with reaction time. Experimental conditions: 0 g/L sodium chloride, 50 mg/L of acetaminophen, amoxicillin, carbamazepine and caffeine; 50 mg/L of copper; 1000 mg/L (stochiometric amount) of hydrogen peroxide; initial pH of 6.

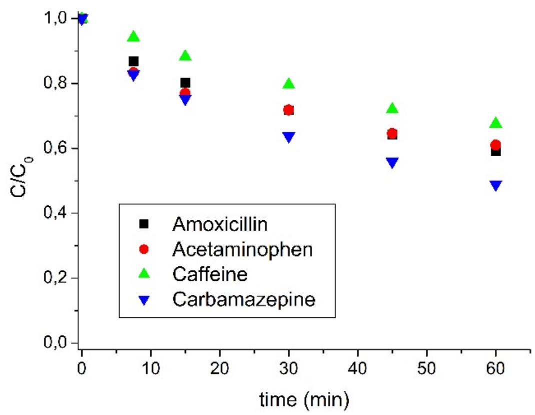

Figure S2. Evolution of the of the pollutants degradation with reaction time. Experimental conditions: 1 g/L sodium chloride, 50 mg/L of acetaminophen, amoxicillin, carbamazepine and caffeine; 50 mg/L of copper; 1000 mg/L (stochiometric amount) of hydrogen peroxide; initial pH of 6.

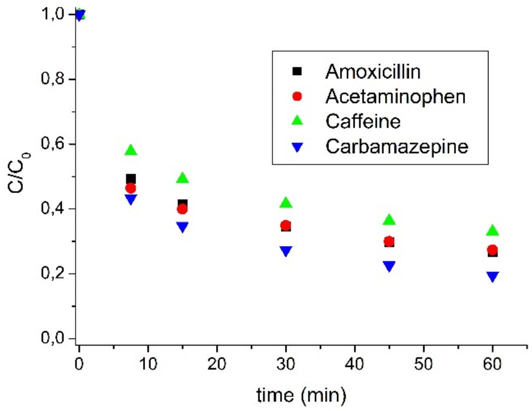

Figure S3. Evolution of the of the pollutants degradation with reaction time. Experimental conditions: 10 g/L sodium chloride, 50 mg/L of acetaminophen, amoxicillin, carbamazepine and caffeine; 50 mg/L of copper; 1000 mg/L (stochiometric amount) of hydrogen peroxide; initial pH of 6.

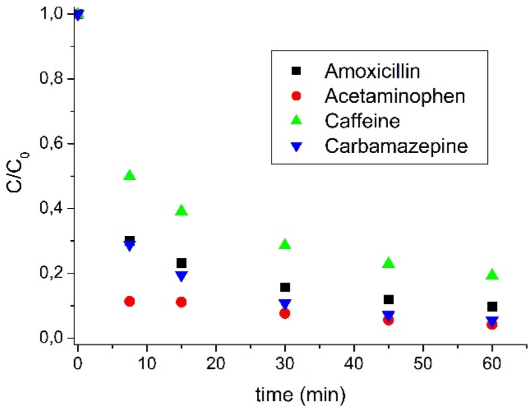

Figure S4. Evolution of the of the pollutants degradation with reaction time. Experimental conditions: 36 g/L sodium chloride, 50 mg/L of acetaminophen, amoxicillin, carbamazepine and caffeine; 50 mg/L of copper; 1000 mg/L (stochiometric amount) of hydrogen peroxide; initial pH of 6.
